# Analyst coverage and real earnings management: Does IFRS adoption matter? UK evidence

**DOI:** 10.1016/j.heliyon.2024.e31890

**Published:** 2024-05-24

**Authors:** Mohammad I. Almaharmeh, Jia Liu, Majd Iskandrani

**Affiliations:** a Accounting Department, Business School, The University of Jordan/Aqaba campus, Jordan; b Professor of Accounting and Finance, Portsmouth Business School, University of Portsmouth, Richmond Building RB6.04, Portland Street, Portsmouth, PO1 3DE, United Kingdom; c Associate professor of finance, Finance department, Bbusiness School, The University of Jordan, Amman, 11942, Jordan

**Keywords:** External corporate governance, Analyst coverage, Real earnings management, IFRS

## Abstract

This study examines the impact of analysts coverage on real earnings management (REM) decisions. The study further investigates the effect of mandatory IFRS adoption on the relationship between analyst coverage and REM. We constructed a sample of UK non-financial listed firms for the period 1997–2021. The results demonstrate that firms followed by a large number of financial analysts record higher levels of earnings management. This result supports the contention that high intensity of analyst coverage imposes extra pressure on firms' managers to meet analysts' earnings per share (EPS) expectations, motivating a higher level of earnings management. Contrary to expectations, the introduction of IFRS fails to strengthen the monitoring role of security analysts on firms' management: rather, managers utilize the inherent flexibility and available discretion in the principles-based IFRS to meet analysts’ benchmarks by means of REM activities. These results are robust after controlling endogeneity.

## 1 Introduction

Existing research on analysts covearge focuses on analysts' role in corporate goverance and financing decisions and studies how analysts control information asymmetry [1]. Neverthless, only a limited number of empirical work has established an association between a firm's real earnings management and security analysts. Previous work has investigated the effect of analysts coverage on accrual earning mangement [2,3]. However, some work has been devoted to examine the effect of analysts coverage on both accrual and real earning management [4,5]. These studies have documented that managers typically make their choices on real earnings management prior to the decisions on accrual earnings management, and that managers prefer real earnings management when they encounter high litigation risk.

Furthermoe, this study has investigated the mediating role played by International Financial Reporting Standards (IFRS) adoption on the association between analysts coveage and real earnings management. Over the past several decades, a single set of global accounting standards, IFRS, was launched to “*bring transparency by enhancing the international comparability and quality of financial information, enabling investors and other market participants to make informed economic decisions*” (IFRS Foundation, 2022).^1^ Therefore, many studies have gauaged the influence of IFRS adoption on earnings mangement [6,7]. For instance, these studies noted that IFRS adoption had no significant change in the pervasiveness of earnings management in Austria or the UK.

Neverthless, there are two prior works linked to our study [8]. examined the effect of IFRS adoption on the association between analyst coverage and earnings management in ASEAN. They documented that analyst coverage affects both accrual and real earnings management. Neverthless, IFRS adoption influences earnings mangement through above the line items, but had no significant effect on earnings management through below the line items.

Notably, there is no difference in the effect of analyst coverage on earnings management in the period and after the adoption of IFRS.

[9], guaged the effect of mandotary adoption of IFRS on the link between analysts coverage and earnings management in the UK context. The author noted a significant negative effect of analyst coverage on earnings management. Moreover, the study documented that mandotory adoption of IFRS does lead to lower earnings management levels. However, IFRS fails to enhance the monitoring role of financial analysts in detecting earnings management.

Our study differs from theirs in the following two important ways. Firstly [8], included in their samples both mandatory and voluntary adopter firms. However, our sample comprises mandatory adopter firms only. Secondly, to measure earnings management, those two studies use both accrual and real earnings mangement. Previous research [10] finds that analyst coverage can effectively constrain earnings management by focusing on accrual earnings management. However, there is little research that explicitly examines the monitoring role of analyst coverage in constraining real earnings management. It is warranted to clarify this issue since analyst coverage may affect accrual and real earnings management differently.

Therefore, our study followed the work of [[11], [12], [13]] and adopted three main measures for manipulating real earnings by managers to increase reported earnings, namely abnormalities in cash flow, cost of production, and discretionary expenses. Based on a sample of 12,615 firm-year observations, on all firms listed on the London Stock Exchange (LSE) over the period 1997–2021. This study finds that firms engage in higher manipulation of real activities when they are followed by a greater number of analysts, indicating that analyst coverage, instead of constraining REM, increases pressure on managers to meet analysts' consensus of predicted firms' performance. Contrary to expectations, IFRS adoption does not enhance the monitoring role of financial analysts in constraining managers' earnings management activities. These results suggest that managers utilize inherent flexibility and available discretion in the principles-based IFRS to meet analysts’ targets through REM activities. Our findings are substantiated by a series of robustness tests and continue to hold after accounting for potential endogeneity concerns.

This study contributes to the literature in the following ways. Firstly, our study extends a growing research stream on the relation between analyst coverage and real activities, by focusing on real earnings management. We measure real activities manipulation in a more comprehensive way, and document more explicit evidence on the association between analyst coverage and real earnings management. Secondly, this study also contributes to the research by adding the effect of mandatory adoption of IFRS on the association between real earnings management and analyst coverage. Our study provides sweeping evidence on this issue in the UK context.

The rest of the paper is organized as follows. Section 2 reviews the literature on real earnings management and analyst coverage. Section 3 develops the hypothesis on the relation between analyst coverage and real earnings management. Section 4 discusses the research design. Section 5 reports the empirical results. Section 6 makes concluding remarks.

## 2 Theoretical framework and prior literature

### 2.1 Theoretical framework

Agency theory posits that agency problems arise when a company's management is separated from its owners, leading managers to act in their self-interest at the owners' expense due to information asymmetry. Financial analysts play a crucial role in mitigating these agency costs by actively monitoring and processing firm-specific information. Analyst coverage, is considered an external governance tool that helps in reducing information asymmetry arising from agency conflicts [3,12,14]. Unlike most investors, analysts possess the ability and knowledge to analyse company disclosures and enjoy privileged access to management, enabling them to observe the firms' activities [15]. Analysts also have a motive to act as monitors, because their reputations as information providers could be comprimised if they base their research reports and recommendations on manipulated earnings figures [16].

Analysts' activities in continuously monitoring managers' behaviour and financial disclosures make them effective monitors of earnings management [17]. find that most firms' financial executives consider stock analysts to be the first or second most influential players after institutional investors in influencing company stock prices [3].argued that since analysts actively engage in information processing and distribution, the financial reporting decisions of managers can be greatly influenced by the intensity of analyst coverage [18]. find that firms voluntary disclosure is affected by the reputation of financial analysts. Moreover [17], find that most firms’ financial executives consider stock analysts to be the first or second most influential players after institutional investors in influencing company stock prices.

Arguably, the greater the analyst coverage, the less likely management will be to engage in earnings manibulation [19]. find that security analysts are among the earliest to monitor and detect firm's fraud. Conssistent with this assertion [20] find that managers manage earning to meet analyst forecast.

### 2.2 Literature review and development of hypotheses

#### 2.2.1 Analyst coverage and earnings management

Earnings management occurs when managers misrepresent their firm's performance in the preparation of financial accounting reports by restructuring the incidence of transactions [[21], [22], [23], [24], [25]]. According to Ref. [21], a firm may choose between real or accruals-based earnings management (AEM) approaches. REM is different from AEM because REM activities rarely violate financial regulations, although they compromise financial reporting quality [26]. Accruals-based methods are founded on acceptable accounting estimates, such as predicting the level of expenses due to bad debts, which do not directly impact cash flows. In comparison, REM techniques can involve modifying normal operational practice to create misleading earnings figures, thus directly affecting cash flow [21]. For this reson shareholders are potentially exposed to greater costs from real earnings management than accruals earnings management [27].

According to Ref. [13], REM relates to activities that managers undertake within a given period to fulfil earnings targets; for example, by aggressively discounting products to demonstrate increased sales volume, thereby avoiding losses and meeting analysts’ earnings expectations. REM differs from AEM in three ways: the former takes place within the current financial year [28]; it is difficult to detect as it can be made to resemble normal activity [29]; and it directly impacts the cash flow of a firm [21]. Thus, real earnings management creates an opportunity for management to alter financial reporting to conceal their myopic behaviour from the public [30].

Recent evidence suggests that managers employ REM to meet analysts’ targets. In a survey of more than 400 US chief executive officers [17], found that executives believe reported earnings, not cash flow, to be the most important performance indicator accessible to outsiders. They also determine that managers prefer to use real activities to manage earnings rather than “cheap” accruals manipulations because this avoids the stigma attached to accounting fraud, as AEM is likely to attract more attention from auditors, regulators, financial analysts or other market players. Similarly [18], suggest that firms voluntary disclosure about earnings forecast is affected by the reputation of analysts following the firms.

In contrast to traditional AEM strategies, where there is a robust accounting framework to assess deviations from acceptable normal practices, real operations are dependent upon the expertise of management, which makes it difficult for external users of financial reports to distinguish between erroneous and optimal decisions [31] [29,32]; [21]. Consistent with this assertion [32], suggest that managers prefer REM because it may be more difficult to detect than AEM, thus entailing lower expected private costs [17,33]. document the use by managers of “harder to detect” REM instead of accruals-based methods after the application of a stricter regulatory mechanism, such as the Sarbanes-Oxley Act (SOX).

The effect of analyst coverage on REM is ambiguous. On the one hand, analyst coverage can be considered an external monitoring tool for exposing managerial misreporting. Financial analysts have strong incentives to achieve a high level of professional performance, which enhances their reputation and compensation, and their efficiency is gauged by the accuracy of their earning forecasts [12]. Therefore, they routinely track corporate information and interact with firms' managers, posing questions about various aspects of earnings' data during profit disclosure conferences [3]. This scrutiny of firms' performance and direct interaction with management improve the analyst's ability to detect earning misreporting, whether through AEM or REM. Thus, a high intensity of analyst coverage helps to reduce opportunistic management disclosure [34].

[19] found that financial analysts are superior to other external governance mechanisms (such as external auditors or the Securities and Exchange Commission (SEC) in detecting misreporting, meaning that financial analysts are also whistle-blowers for accounting manipulation or fraud [35]. [3,10], suggest that the financial analyst acts as an external monitor of a firm's financial reporting. In addition, investors in the options market recognise security analysts as important information intermediaries and monitors, and recognise that their oversight influences stock crash risk [36] [37];. Further, research demonstrates that analysts monitor companies' research and development investment [38], cost structure [39], and operational decisions [40], and thus may prevent managers from undertaking substantive actions to manipulate earnings. Therefore, a high intensity of analyst coverage is likely to impede managers' REM activities such that they undertake less REM than firms with lower analyst coverage.

Alternatively, it can be argued that a high level of security scrutiny may lead to extra pressure on management to increase real earnings manipulation. Consistent with this assumption [38],), report that firms covered by a high number of financial analysts generate fewer patents or generate patents with a lower significant impact. Additionally, in firms followed by more analysts, managers may have greater incentives to meet or exceed earnings expectations because unfavourable news of missing earning benchmarks will be incorporated more rapidly into stock prices [12]. Failing to meet the analysts’ consensus leads to negative market reactions [41], e.g., a decline in the annual bonus of managers [42]; and a greater likelihood of managerial turnover [[42], [43], [44]]. Consistent with assertion (20found that firm management perform earnings management activities to meet or beat analyst earninigs forecast., as AEM is easier to detect than REM, managers monitored by a greater number of financial analysts are more likely to use REM than AEM [3,28].

Overall, the negative effect of analysts' coverage on REM, resulting from their monitoring of firms’ performance can be offset by the positive effect of the pressure placed by financial analysts on managers to meet or beat their earning benchmarks. Based on the foregoing discussions, we propose our first hypothesis below.

H1 Analyst coverage has a significant influence on real earnings management.

#### 2.2.2 The effect of mandatory IFRS adoption

In the UK setting, UK GAAP was “high quality” and the UK stock market featured sophisticated market participants and information exchange mechanisms. Where the UK is a common law country characterised by strong investor's protection regimes. However, in the 2006 Review of Narrative Reporting by UK Listed Companies, the Accounting Standards Board (ASB) reported that, in previous years, UK firms generally did not disclose any non-financial key performance indicators or risk-related information with significant decision-usefulness. In the 2007 Narrative Reporting Survey, PricewaterhouseCoopers has shown that, in general, UK firms did not present adequate quantitative financial information. The 2008 Black Sun research reported that only a small percentage of firms communicated sufficiently their business objectives and strategy or linked their narrative disclosures to numerical data.

Prior research affirms the proposition that the mandatory adoption of International Financial Reporting Standards (IFRS) significantly enhances the quality of financial disclosures. Contemporary evidence, such as studies by Refs. [45,46], and [47], establishes a positive correlation between IFRS adoption and improved accounting and earnings quality. Additionally, contributions by Refs. [48,49] emphasize the heightened value relevance of accounting numbers following the implementation of IFRS.

Recent studies, such as those conducted by Refs. [50,51], further support the positive impact of IFRS adoption on financial reporting quality [50]. argues that IFRS adoption reduces income smoothing and enhances the extent of conditional conservatism. [51], regression results showed a significant negative effect of IFRS adoption on earnings management, thus indicating an improvement in the financial reporting quality.

The adoption of IFRS is anticipated to enhance the effectiveness of financial analysts in preventing earnings management, driven by several factors. Firstly, IFRS adoption is associated with a more transparent and higher-quality information environment, as evidenced in work by Refs. [52,53], and [54]. This heightened transparency empowers financial analysts by providing them with improved monitoring capabilities. Secondly, IFRS adoption reduces information processing costs through enhanced comparability, as demonstrated in studies by Refs. [55,56], and [57]. Analysts can leverage this improvement to access diverse information sources, enabling them to verify the accuracy of accounting data disclosed by the companies under their purview, as highlighted by Ref. [16]. Lastly, the likelihood of firms being overseen by financial analysts increases with IFRS adoption due to heightened foreign investment, according to studies by Refs. [58,59], and [60]. Consequently, the adoption of IFRS is expected to facilitate financial analysts' monitoring of firms, as failure to do so may result in substantial losses attributed to analysts' mistrust.[1]

Based on these discussions, we propose our second hypothesis below.

H2 Mandatory IFRS adoption facilitates the detection of real earnings management by financial analysts and thus reduces firms' real earnings management.

## 3 Research design

### 3.1 Sample development

Our initial sample for the analysis includes all firms listed on the London Stock Exchange (LSE) over the period 1997–2021. We obtain financial accounting information from DataStream to measure REM and control variables. Data on analysts' EPS forecasts, to calculate the analyst coverage variable, were collected from the Institutional Brokers’ Estimate System (I/B/E/S) database. Consistent with the literature (e.g. Refs. [61,62], and [63], our study focuses on one-year-ahead earnings forecasts. After the exclusion of observations with missing data and firms in the financial sector with SIC 6000–6999, the final sample comprises 12,615 firm-year observations for our analysis.

Our study covers a long period, following the recommendation of [64], who observe that a longer duration provides more representative results and conclusions. Our sample starts in 1997, as there is concern over the quality of I/B/E/S data for the early 1980s when there were fewer analysts than in the 1990s.

We examine a single country, namely the UK, to control for factors that may affect the results, such as accounting disclosure requirements, stock listing requirements, the regulatory environment, and market microstructures, as suggested by Refs. [65,66]. In addition, choosing a single country helps to minimise heterogeneity and cross-countries differences that may affect explanatory variables [67]. Furthermore, we examine UK data for its distinctiveness. According to Ref. [68], the financial reporting environment in the UK is considered to be very shareholder-oriented, providing an ideal setting for a better assessment of the impact of IFRS adoption on the relationship between stock analysts and REM.

Table 1 reports the characteristics of the sample. Panel (A) presents the number of sample firms for each year of the sample period. Panel (B) classifies the sample by the two-digit SIC industry codes: business services (19.56 %), engineering, accounting, research, and management services (7.35 %), oil and gas extraction (6.48 %), chemicals and allied products (5.98 %), and metal and mining (5.6 %) are the most representative industries.

**Table 1:** Sample breakdown.

Panel A: by year		
Year	Frequency	Percent
1997	102	0.81
1998	128	1.0
1999	135	1.07
2000	181	1.43
2001	269	2.13
2002	303	2.40
2003	340	2.70
2004	369	2.93
2005	410	3.25
2006	384	3.04
2007	411	3.26
2008	517	4.10
2009	616	4.88
2010	662	5.25
2011	701	5.56
2012	678	5.37
2013	681	5.40
2014	711	5.64
2015	722	5.72
2016	729	5.78
2017	740	5.87
2018	710	5.63
2019	709	5.62
2020	678	5.37
2021	729	5.78
Total	12,615	100

Panel B: By industry			
Two_digit SIC code	Industry description	Frequency	Percent (%)
10	Metal mining	706	5.60
13	Oil and gas extraction	818	6.48
15	Construction- general contractors & operative builders	312	2.47
20	Food products	410	3.25
27	Printing, publishing and allied industries	420	3.33
28	Chemicals and allied products	755	5.98
35	Industrial machinery and equipment	514	4.07
36	Electrical and electronic equipment	624	4.95
38	Instruments and related products	500	3.96
48	Communications	359	2.85
50	Wholesale trade – durable goods	327	2.59
58	Eating and drinking places	350	2.77
73	Business services	2468	19.56
87	Engineering, accounting, research, and management services	927	7.35
Others		3125	24.79
Total		12,615	100

### 3.2 Measurement of variables

#### 3.2.1 Earnings management measures

We adopt three main measures for manipulating real earnings by managers to increase reported earnings, namely abnormalities in cash flow, cost of production, and discretionary expenses, as in Ref. [13]. A firm may temporarily increase its sales volumes through discounting the price of its products or services, or through making improved credit offerings to increase sales revenue. However, these increases in sales revenue are likely to disappear once the firm reverts to its former prices [31].

Alternatively, a firm may focus on overproducing (underproducing) production units with the intention of decreasing (increasing) fixed costs per unit, with a decreased (increased) cost for product sales, resulting in increased (decreased) earnings [31,69]. show that managers use overproduction to inflate short-term profitability at the expense of long-term value. The overproduction spreads fixed costs over all units produced because of absorption costing, leading to a lower cost of goods sold and higher current-period earnings.

Additionally, discretionary expenses, including advertising, sales, research and development, administration and general costs, are usually classified as expenses when they are incurred, and a manager can deliberately decrease (increase) such costs to increase (decrease) current year earnings [31] [69]; [40].

In line with previous studies on earnings management [70] [32]; [31]; [40,71,72];, we apply [13] three models for measuring estimated REM. Operational cash flows, CFO, are estimated using per-industry, per-year cross-sectional regressions [2],[3]:

(1) $$\frac{CFO}{LTA}=\alpha_{0}\left(\frac{1}{LTA}\right)+\alpha_{1}\left(\frac{SALES}{LTA}\right)+\alpha_{2}\left(\frac{\Delta SALES}{LTA}\right)+\varepsilon_{t}$$

In which CFO represents operational cash flow and LTA = lagged total assets. SALES represents a company's sales in year *t*, and ΔSALES represents sales change. Estimated coefficients of Model [1] are used to measure the normal CFO, while normal CFO is deducted from actual CFO to calculate abnormal CFO. Upward (downward) manipulation of earnings by a firm will result in negative (positive) residual values. Therefore, significantly positive or negative (CFO*)* suggests extensive earnings manipulation.

The second measure used to identify REM is an abnormality in discretionary expenses (DIS_EX). This is derived by summing research and development, sales, administration and general expenses. We apply per-industry per-year cross-sectional regressions for estimation of DIS_EX, which is specified as follows:

(2) $$\frac{{DIS\_EX}_{t}}{LTA}=\alpha_{0}+\alpha_{1}\left(\frac{1}{LTA}\right)+\beta_{1}\left(\frac{{SALES}_{t-1}}{LTA}\right)+\varepsilon_{t}$$

in which DIS_EX represents discretionary expenses, and ${SALES}_{t-1}$ represents lagged sales. Abnormal discretionary expenses, DIS_EX is defined as the difference between the values given by Model [2] and the actual values. Similar to *CFO*, greater residual value for DIS_EX suggests greater levels of earnings management.

Production costs, PROD, are derived by summing costs for products sold with inventory change in year t. Model(3)is specified to estimate the normal cost of production:

(3) $$\frac{{PROD}_{t}}{LTA}=\alpha_{0}+\alpha_{1}\left(\frac{1}{LTA}\right)+\beta_{1}\left(\frac{{SALES}_{t}}{LTA}\right)+\beta_{2}\left(\frac{\Delta {SALES}_{t}}{LTA}\right)+\beta_{3}\left(\frac{\Delta {SALES}_{t-1}}{LTA}\right)+\varepsilon_{t}$$

in which PROD = production costs. The dependent variables are determined as described earlier. The residual value derived from Model [3] is applied to measure abnormal production (PROD).

Next, we derive three measures which combine the previous measures to investigate REM; that is, values from *CFO, PROD and DIS_EX.,* in line with [21,31,71,73]. Calculation of the combined measures is described below:

(4) *REM1* = *PROD* + *DIS_EXP*

(5) *REM2* = *CFO* + *DIS_EX*

(6) *REM3 = CFO* + *PROD* + *DIS_EX*

Thus, greater values for these measures suggest that REM is more likely to have occurred.

#### 3.2.2 Measurement of analyst coverage

Following [9], we adopt the number of analysts who have made one-year ahead earnings forecasts or recommendations in any given fiscal year to proxy for the intensity of financial analyst coverage, *FOLL*.

### 3.3 Empirical models

To examine the effect of analyst coverage on REM, we estimate the following model:

(7) $${REM}_{i,t}=\alpha_{0}+\beta_{1}{FOLL}_{i,t}+\beta_{2}{LEV}_{i,t}+\beta_{3}{M/B}_{i,t}+\beta_{4}{SIZE}_{i,t}+\beta_{5}{ROA}_{i,t}+\varepsilon_{i,t}$$

In Model [7], if analyst coverage is applied as an external governance tool, with effective monitoring of the managers, it will restrain REM activities and lead to a significant negative coefficient on FOLL. Conversely, if a higher level of analyst coverage means that management feels more pressurised to achieve or exceed earnings consensus, then positive value on FOLL is expected.

To investigate the impact of making IFRS obligatory on REM's association with analyst coverage: *IFRS* and IFRS*FOLL, we have added two variables to model [7].

(8) $${REM}_{i,t}=\alpha_{0}+\beta_{1}{FOLL}_{i,t}+\beta_{2}{IFRS}_{i,t}+\beta_{3\;}IFRS*FOLL+\beta_{4}{LEV}_{i,t}+\beta_{5}{M/B}_{i,t}+\beta_{6}{SIZE}_{i,t}+\beta_{7}{ROA}_{i,t}++\varepsilon_{i,t}$$

Where *IFRS* is a dummy variable, which equals one if the firm prepares its financial statements under IFRS, and zero otherwise. Since the adoption of IFRS is mandotry for all UK firms after 2005, this variable will equal 0 if the year is 2004 or before and equal to 1 from 2005 or after. It is expected that the reductions in information processing costs after adopting IFRS will facilitate the governance role that analysts play in monitoring management, and lead to a statistically significant negative coefficient for the interaction term, IFRS*FOLL variable.

We control for a set of variables, as identified in other studies, which may influence REM: financial leverage, which is measured by total liabilities divided by total assets (LEV); market-to-book ratio (M/B), return on assets (*ROA*), and size of the company measured by total assets in the natural log form (SIZE),.

[74], shows that financial leverage and earnings management are positively related. A larger firm may have significant advantages, with reduced pressure to exaggerate its financial performance [75]. In addition [76], argue that larger companies tend to apply internal controls more effectively than smaller ones, which potentially reduces earnings management. Furthermore, large audit firms generally work with larger companies, engendering higher quality financial reporting and enhancing detection and prevention of earnings management practices [76]. Further, a higher level of leverage increases monitoring by banks and creditors, thus reducing the opportunity for management to manipulate earnings [77] [78,79]; [80]. reveals that investors find it difficult to estimate the value of high-growth firms due to the difficulty in valuing their growth opportunities, which in turn provides managers with more flexibility to manage reported earnings and mislead investors. Return on assets (ROA) controls for firm's profitability. Prior research suggest a positive relation between financial profitability and real earnings management. [81,82], suggest that managers use real activities to improve current period's earnings and signal better future performance through meeting analysts earnings benchmarks. Winsorisation is carried out for each continuous variable for the 1st and 99th percentile. The final sample for the study contains 12,615 firm-year observations from 1401 UK companies.

## 4 Descriptive statistics and regression results

### 4.1 Descriptive statistics

Table 2 reports descriptive statistics for all variables used in the analyses. The means (medians) of real earnings quality measures: *DIS_EX, PROD,* and *CFO*, are 0.203 (152), 0.202 (0.143) and 0.141 (0.111), respectively these values are relatively conssistent with the values reported by Refs. [83,84], and [40]. The sample firms are, on average, followed by between one to forty analysts, with 75 % followed by fewer than nine. In respect of the control variables (*SIZE*, *ROA*, *LEV, M/B*), the statistics show a significant level of dispersion in their values, reflecting the heterogeneity of the firm-year sample. The mean value of M/B value is 2.92 which is consistent with that reported by Ref. [81].

**Table 2:** Descriptive statistics.

Variable name	P25	Mean	Median	P75	Std. Dev.	Min	Max
DIS_EX	0.062	0.203	0.152	0.303	0.171	0.0001	0.652
PROD	0.059	0.202	0.143	0.296	0.183	0.0001	0.823
CFO	0.059	0.141	0.111	0.186	0.116	0.0001	0.513
IFRS	0	0.692	1	1	0.462	0	1
Analysts-following (*FOLL*)	1	6.222	3	9	6.838	1	40
Financial leverage (*LEV*)	0.091	0.20	0.172	0.260	0.185	0.0001	3.714
Growth Opportunity (M/B)	1.07	2.924	1.870	3.392	4.614	−12.666	30.326
Firm size (log-total asset) (*SIZE*)	10.235	11.879	11.646	13.339	2.291	0	20.057
ROA	−0.025	−0.019	0.040	0.087	0.0244	−1.351	0.974

Notes: this table provides descriptive statistics for the full sample variables of interest. The sample consists of 12,615 firm-year observations gathered from 1401 UK firms for the period from 1997 to 2021.

### 4.2 Correlation analysis

Table 3 provides the Pearson correlation matrix between REM measures and the independent variables. The number of financial analysts who follow a firm exhibits a significant correlation with two of the REM measures: abnormal discretionary expenses and abnormal cash flow from operations while showing no correlation with abnormal production levels. This may suggest that high intensity of analyst coverage leads to fewer REM activities.

**Table 3:** Pearson correlation matrix.

Variable	CFO	DIS_EX	PROD	IFRS	SIZE	ROA	LEV.	M/B	FOLL
CFO	1								
DIS_EX	0.3194***	1							
PROD	0.1076***	0.3677***	1						
IFRS	0.0361***	−0.0036	−0.0375***	1					
SIZE	−0.0138	−0.0861***	−0.0522***	0.0862***	1				
ROA	−0.0083	−0.0051	0.024***	−0.0141	03519***	1			
LEV	0.0062	−0.0519999	−0.0506***	0.0097	0.1294***	−0.1209***	1		
M/B	0.0288***	0.0385***	0.0105	−0.0871***	−0.0637	−0.0976***	−0.0703***	1	
FOLL	0.0328***	−0.018**	−0.01333	0.1133***	0.764***	0.211***	0.1249***	0.0553***	1

Notes: this table provides the results of Peason correlation for the full sample variables of interest. The sample consists of 12,615 firm-year observations gathered from 1401 UK firms for the period from 1997 to 2021. *Significance at 10 % level (two-tailed tests). **Significance at 5 % level (two-tailed tests). ***Significance at 1 % level (two-tailed tests).

The correlations between the three measures of REM are positive and significant, which indicates that firms simultaneously use different strategies for real activities' manipulation to achieve their earnings objectives [21]. Furthermore, the negative correlations between SIZE and the three measures of REM suggest that larger firms are less likely to engage in REM activities. The LEV variable reveals a significant negative correlation with *DIS_EX* and *PROD*, signifying that highly leveraged firms might be exposed to greater scrutiny and monitoring from creditors and bankers, which limits managers’ opportunities to engage in earnings management activities [[77], [78], [79]].

The highest correlation coefficient between FOLL and SIZE (0.76) may imply that financial analysts prefer to follow larger, rather than smaller firms. All other correlation coefficients present values of less than 0.80, indicating that multicollinearity is not an issue in this study. In addition, we perform variance inflation factor test (VIF) as another test for multicollinearity. Prior literature documented that if VIF test is less than 10 that means there is no multicollinearity problem. The untabulated results of VIF test suggest that the collinearity is not an issue in this research.

### 4.3 Results and analysis for multiple regression

Table 4 reports the results of model [7] for the three measures of REM across the entire sample. The results exhibit a significant positive association between financial analysts and the measures of REM: *CFO, DIS_EX*, and *PROD* (t-statistics = 6.78, 8.37, and 5.

**Table 4:** Regression results of the effect of analyst coverage on REM.

VARIABLE	*CFO*	*DIS_EX*	*PROD*
*FOLL*	0.002	0.0029	0.0019
	(6.78) ***	(8.37) ***	(5.35) ***
*LEV*	0.0048	−0.0349	−0.0381
	(0.74)	(-3.79) ***	(-3.95) ***
*M/B*	0.0005	0.0008	0.0001
	(1.90) *	(2.2) **	(0.08)
*SIZE*	−0.0046	−0.0134	−0.0095
	(-6.12) ***	(-12.29) ***	(-8.08) ***
*ROA*	0.0027	0.0217	0.0343
	(0.55)	(3.12) ***	(4.81) ***
*Intercept*	0.183	0.3485	0.3102
	(22.30) ***	(29.81) ***	(24.77) ***

Notes: Table 4 presents the OLS multivariate regression results from the estimation of Model [7]. The first column presents the explanatory variables. *SIZE* is the natural log of the firm's total assets at the end of the year. *ROA* is operating income divided by total assets. *LEV* is total liabilities divided by total assets. *M/B*is the market value of equity to book value of equity ratio. *FOLL* is the number of analysts providing one-year earnings per share (EPS) forecast for a firm. The second, third, and fourth columns present the coefficient and t-values (in parentheses) for real earnings management proxies. *CFO* is abnormal cash flow from operation; *DIS_EX* is the abnormal discretionary expense; *PROD* is the abnormal production costs. T –values are heteroscedasticity robust clustering at the firm level. Here, *, **, and *** represent 10, 5, and 1 % levels of significance respectively for the two-tailed test.

35, respectively). This positive effect of analyst coverage on all REM measures demonstrates that analyst coverage imposes extra pressure on managers to meet analysts' expectations through real activities manipulation by way of increased sales, overproduction, and discretionary expenses.These results provide strong support for hypothesis 1, that high-intensity of analyst coverage places greater pressure on managers to meet their earnings expectations by engaging in REM. This positive effect can be explained by that firms' managers who fail to meet analysts’ forecasts frequently suffer serious consequences, such as lower compensation and a greater possibility of being dismissed [43,44].

These results are consistent with the findings of [12,40], suggesting that more analyst coverage puts pressure on managers to meet analysts' forecasts, leading to increased earnings management activities. However, they are contrary to the findings of [3,16], who find that analyst coverage plays an effective role in constraining AEM. Our findings suggest that external governance measures may not effectively mitigate the agency problem; instead, might exacerbate this problem. This outcome may arise because a company's managers possess more information than the owners, leading them to prioritise self-interest at the expense of the owners, through managing the earnings to meet analyst expectations.

Regarding the control variables, SIZE demonstrates a significantly negative effect on measures of REM, suggesting that large firms engage in fewer earnings management activities. Moreover, highly leveraged firms are less prone to managing earnings through their discretionary expenses and discretionary production costs. Consistent with the results of [81,82]; ROA records a significant positive effect on *DIS_EX* and *PROD*. The firm grouth apportunity (M/B) records a significant positive effect *CFO* and *DIS_EX.* This result corroborate the results of [40], and suggests that the difficulty in valuing the growth opportunities in high grouth firms provides managers with more flexibility to manage reported earnings.

Table 5 reports the results of the impact of analysts’ coverage on REM, which is represented by three composite measures of real activities manipulation as used in Refs. [21,31], and defined in Models [4], *(*5) and *(*6).

**Table 5:** Regression results of the effect of analyst coverage on additional aggregate measures of real earnings management.

VARIABLE	*REM1*	*REM2*	*REM3*
*FOLL*	0.0048	0.0046	0.0065
	(8.48) ***	(9.29) ***	(9.5) ***
*LEV*	−0.0730	−0.0301	−0.0682
	(-4.68) ***	(-2.37) **	(-3.59) ***
*M/B*	0.0008	0.0012	0.0013
	(1.3100)	(2.59) ***	(1.8) *
*SIZE*	−0.0228	−0.0180	−0.0275
	(-12.25) ***	(-11.81) ***	(-12.43) ***
*ROA*	0.0560	0.0244	0.0587
	(4.87) ***	(2.52) ***	(4.35) ***
*Intercept*	0.6587	0.5318	0.8420
	(32.73) ***	(32.46) ***	(35.36) ***

Notes: Table 5 presents the OLS multivariate regression results from the estimation of Model [7]. The first column presents the explanatory variables. *SIZE* is the natural log of the firm's total assets at the end of the year. *ROA* is operating income divided by total assets. *LEV* is total liabilities divided by total assets. *M/B*is the market value of equity to book value of equity ratio. *FOLL* is the number of analysts providing one-year earnings per share (EPS) forecasts for a firm. The second, third, and fourth columns present the coefficient and t-values (in parentheses) for aggregate real earnings management proxies. *REM1* = *PROD* + *DIS_EXP, REM2 = CFO + DIS_EX,* and *REM3 = CFO* + *PROD* + *DIS_EX*. T–values are heteroscedasticity robust clustering at the firm level. Here, *, **, and *** represent 10, 5, and 1 % levels of significance respectively for the two-tailed test.

The regression results of the three combined measures of REM, namely *REM1, REM2, and REM3*, are presented in Table 5. The results reveal that all coefficients of the analyst variable are positive and significant (at the 1 % level). These findings are consistent with the argument that a high intensity of analyst coverage puts extra pressure on firms' management to meet analysts’ expectations, leading to increased REM. In particular, the significant and positive coefficients of the three measures indicate that, for firms with a high analyst coverage, management is more likely to increase earnings through higher production costs and cutting discretionary expenses (*REM1*); sales manipulation and cutting discretionary expenses (*REM2*); and through the composite measure of REM (abnormal sales figures, abnormal production costs, and abnormal discretionary expenses combined). The evidence supports the finding of [78] that external governance mechanisms may stimulate earnings management.

In respect of the control variables, the signs of their coefficients are as expected. Larger firms and highly leveraged firms have significantly lower REM levels, while more profitable firms and firms with high growth opportunities record positive effects of REM, which are consistent with [12].

In summary, the results for all six measures of REM (*CFO, DIS_EX, PROD, REM1, REM2, and REM3*) demonstrate that a high intensity of analyst coverage leads to high levels of REM. This result does not agree with [3], who finds a negative relationship between analyst coverage and AEM. Our finding supports the view that a high intensity of analyst coverage puts extra pressure on managers to meet analysts' EPS consensus. We argue that managers manipulate earnings to avoid the unfavourable consequences of failing to meet analysts’ earning expectations, such as lower compensation [85] and a greater possibility of dismissal [43,44].

### 4.4 The effect of IFRS adoption

To examine the effect of IFRS adoption on the relationship between analyst coverage and earnings management, we incorporate an interaction term between analyst coverage and IFRS adoption, IFRS*FOLL, to the regression model. Table 6 reports the multiple regression results of the interactions. The results provide mixed evidence of the effect of mandatory IFRS adoption on REM measures. The *IFRS* variable records a significant positive effect on *CFO* (t = 2.61), and no effect on *DIS_EX* (t = −1.59) and negative effect on *PROD* (t = −4.69) and. Therefore, the net effect of IFRS adoption on earnings management is ambiguous, and further evidence is needed to determine its impact.

Concerning the interaction term variable (IFRS*FOLL), the coefficient of this variable measures the moderating effect of mandatory IFRS adoption on the relationship between analyst coverage and REM. The results show that the coefficient of the IFRS*FOLL variable is positive at a 5 % level in the case of *DIS_EX* and *PROD*. This result suggest that, firm's managers may utilize the inherent flexibility and the available discretions of the principles-based IFRS to manipulate earnings through discretionary expenses and discretionary production costs, which in turn complicates the task for financial analysts in detecting potential earnings management. The principles-based nature of IFRS allows managers to exercise judgment in financial reporting, tailoring statements to meet analysts' expectations. However, this flexibility introduces complexity for analysts, as interpretations and application of standards may vary across entities. Consequently, it complicates the task for financial analysts, making the detection of potential earnings management a more complex task.

In summary, our results clearly show that after the *IFRS* and IFRS*FOLL variables are added to the regression model, the effect of analyst coverage on REM remains significantly positive for *all three measures of REM*; and such a significant positive effect of security analysts on REM is not reduced by the mandatory adoption of IFRS. Therefore, we conclude that high intensity of analyst coverage encourages earnings management, even in the presence of high-quality and more transparent accounting reporting standards.

### 4.5 Endogeneity issues

As financial analysts may be willing to follow firms with high or low levels of accounting quality [12], it is difficult to ignore reverse causality or endogeneity problems. To address this serious endogeneity issue, we implement a two-stage least square model (2SLS). The first-stage model is as follows:

9 Analyst=α0+β1SIZE+β2ROA+β3M/B+ε

In the second stage, we use the fitted value of analyst coverage (*P-analyst*), extracted from Model 9, in the regression instead of the observed value. Table 7, Table 8 and 8 represent the results of the second-stage regression. The results suggest a positive relationship between the fitted value of analyst coverage and all the measures of REM. In particular, Table 7 shows that the fitted value of analyst coverage (*P-Analyst*) records a significant positive effect on the absolute value of *CFO, DIS_EX,* and *PROD* (t-statistics = 2.32, 3.93, and 3.17 respectively).

**Table 6:** Regression results of the effect of IFRS adoption on the relationship between analyst coverage and REM.

VARIABLE	CFO	DIS-EX	PROD
*FOLL*	0.0014	0.0017	0.0008
	(4.16) ***	(3.16) ***	(1.29)
*IFRS*	0.0076	−0.0071	−0.0224
	(2.61) ***	(-1.59)	(-4.69) ***
IFRS*FOLL	0.0002	0.0014	0.0016
	(0.65)	(2.65) ***	(2.74) ***
*LEV*	0.0052	−0.0351	−0.0390
	(0.8100)	(-3.8)***	(-4.05) ***
*M/B*	0.0006	0.0008	−0.0001
	(2.23) **	(2.13) **	(-0.36)
*SIZE*	−0.0046	−0.0131	−0.0092
	(-6.05) ***	(-12.01) ***	(-7.81) ***
*ROA*	0.0036	0.0213	0.0324
	(0.7100)	(3.05) ***	(4.54) ***
INTERCEPT	0.1778	0.3512	0.3230
	(21.35) ***	(29.42) ***	(25.5) ***

Notes: Table 6 presents the OLS multivariate regression results from the estimation of Model [8]. The first column presents the explanatory variables. *SIZE* is the natural log of the firm's total assets at the end of the year. *ROA* is operating income divided by total assets. *LEV* is total liabilities divided by total assets. *M/B* is the market value of equity to book value of equity ratio. *FOLL* is the number of analysts providing one-year earnings per share (EPS) forecast for a firm. *IFRS* is a dummy variable; IFRS = 1 if the firm uses IFRS, and IFRS = 0 if the firm applies other accounting standards. The second, third, and fourth columns present the coefficient and t-values (in parentheses) for real earnings management proxies. *CFO* is abnormal cash flow from operation; *DIS_EX* is the abnormal discretionary expense; *PROD* is the abnormal production costs. T–values are heteroscedasticity robust clustering at the firm level. Here, *, **, and *** represent 10, 5, and 1 % levels of significance respectively for the two-tailed test.

**Table 7:** Regression results on the effect of *P_Analyst* coverage on REM measures.

VARIABLE	CFO	DIS-EX	PROD
*P_ANALYST*	0.0289	0.0740	0.0764
	(2.00)**	(3.72)***	(3.36)***
*LEV*	0.0047	−0.0364	−0.0405
	(0.7200)	(-3.79)***	(-4.04)***
*M/B*	0.0039	−0.0106	−0.0119
	(-1.68)*	(-3.33)***	(-3.24)***
*SIZE*	−0.0690	−0.1811	−0.1853
	(-2.02)**	(-3.86)***	(-3.45)***
*ROA*	0.0460	0.1345	0.1525
	(1.99)**	(4.16)***	(4.15)***
*Constant*	0.7916	1.9349	1.9734
	(2.45)***	(4.36)***	(3.89)***

Notes: Table 7 presents the second stage regression results. The first column presents the explanatory variables. *SIZE* is the natural log of the firm's total assets at the end of the year. *ROA* is operating income divided by total assets. *LEV* is total liabilities divided by total assets. *M/B* is the market value of equity to book value of equity ratio. *P_Analysts* is the predicted value of *Analyst* as estimated using Model (9). The second, third, and fourth columns present the coefficient and t-values (in parentheses) for real earnings management proxies. *CFO* is abnormal cash flow from operation; *DIS_EX* is abnormal discretionary expense; *PROD* is abnormal production costs. T–values are heteroscedasticity robust clustering at the firm level. Here, *, **, and *** represent 10, 5, and 1 % levels of significance respectively for the two-tailed test.

**Table 8:** Regression results of the effect of P_Analyst coverage on the compound REM measures.

VARIABLE	REM1	REM2	REM3
*P_ANALYST*	0.1504	0.1029	0.1793
	(4.05)***	(3.69)***	(4.21)***
*LEV.*	−0.0769	−0.0317	−0.0722
	(-4.66)***	(-2.4)**	(-3.62)***
*M/B*	−0.0225	−0.0145	−0.0264
	(-3.76)***	(-3.24)***	(-3.85)***
*SIZE*	−0.3665	−0.2501	−0.4354
	(-4.18)***	(-3.8)***	(-4.33)***
*ROA*	0.2870	0.1805	0.3330
	(4.78)***	(3.98)***	(4.81)***
*Constant*	3.9083	2.7265	4.6999
	(4.71)***	(4.380)***	(4.94)***

Notes: Table 8 presents the second-stage regression results The first column presents the explanatory variables. *SIZE* is the natural log of the firm's total assets at the end of the year. *ROA* is operating income divided by total assets. *LEV* is total liabilities divided by total assets. *M/B* is the market value of equity to book value of equity ratio. *P_Analysts* is the predicted value of *Analyst* as estimated using Model *(*9). The second, third, and fourth columns present the coefficient and t-values (in parentheses) for aggregate real earnings management proxies.*REM1* = *PROD* + *DIS_EXP, REM2 = CFO + DIS_EX,* and *REM3 = CFO* + *PROD* + *DIS_EX*. T–values are heteroscedasticity robust clustering at the firm level. Here, *, **, and *** represent 10, 5, and 1 % levels of significance respectively for the two-tailed test.

In addition*, P-Analyst* records a significant positive effect on the three compound measures of REM. The t-statistics values for *REM1, REM2, and REM3* are 4.06, 3.99, and 4.32, respectively. These results corroborate the main results and suggest that external governance mechanisms, as measured by a high level of analyst coverage, may stimulate earnings management by imposing extra pressure on managers to meet analysts’ expectations.

These results are in line with the suggestions of [28,40], that firms followed by a greater number of security analysts are more likely to use harder-to-detect REM than traditional AEM methods, and thus incur lower expected private costs.

## 5 Conclusion

This study examines the role of information intermediaries in corporate governance by investigating the effect of analyst coverage on REM. In particular, it explores the effect of analyst coverage on REM as measured by abnormal cash flows from operations, abnormal production costs, and abnormal discretionary expenses. It further investigates the effect of IFRS adoption on the relationship between security analysts’ coverage and REM.

Preceding studies have examined this association based on US markets, providing inconclusive evidence. Our study analyses the UK market, shedding new light on this area, because this market exhibits strong investor protection, high-quality accounting and high stock market liquidity. Moreover, the absence of IFRS adoption in the UK before 2005 makes it an appropriate research setting to examine the effects of mandatory IFRS adoption on the relationship between analyst coverage and earnings management. We have examined this relationship based on 12,615 firm-year observations of 1401 UK firms from 1997 to 2021.

Several interesting findings emerge. Firms engage in more manipulation of real activities when they are followed by a greater number of analysts, indicating that analyst coverage, instead of constraining REM, increases pressure on managers to meet analysts' consensus of predicted firms' performance. Contrary to expectations, IFRS adoption does not enhance the monitoring role of financial analysts in constraining managers' earnings management activities. These results suggest that managers utilize inherent flexibility and available discretion in the principles-based IFRS to meet analysts’ targets through REM activities. Our findings are substantiated by a series of robustness tests and continue to hold after accounting for potential endogeneity concerns.

This paper is one of the first to examine the effect of mandatory IFRS adoption on the association between analyst coverage and REM and extends previous research on the economic consequences of the regulators’ decision to mandate IFRS in general, and on the relationship between analyst coverage and REM in particular. In addition, this research contributes to the earnings management literature, by providing evidence about the determinants of earnings management decision. Understadning the determinants of earning management can contribute to mitigating the agency problem.

Our findings provide insights for regulatory bodies and managers. The data demonstrates the potential for managers' manipulation of earnings through real activity, with the aim of meeting earnings targets set by analysts. Importantly, these conclusions imply that, while the high intensity of analyst coverage associated with reduced earnings managemet from accruals, as seen by Ref. [86] [87], and [3], managers may respond to the increased pressure to meet analysts' expectations by using hard-to-detect real activities' earninigs manipulation in ways that depart from expected business behaviour, imposing financial costs on the firm [17]. The view that the intensity of analyst coverage imposes extra pressure on managers has testable implications for research on the dark side of analyst coverage. Understadning the determinants of earning management can contribute to mitigating the agency problem. In addition, the fact that among the firms that adopted IFRS, those that were followed by a higher number of financial analysts engaged in higher REM activities, suggesting that managers use the discretion and accounting choices available in IFRS to meet analysts’ forecasts, which provides a clear message to regulators and policymakers to reassess their decision to mandate IFRS, by examining the obtained benefits and costs of IFRS adoption.

As with any other research, this study is not without limitations, so the results should not be interpreted without caveats. These limitations provide excellent opportunities to support future researches engagement in addressing these limitations. The fact that this study collects its data from one country, the UK, may limit the generalisation of the results even if it can be applicable to the countries that have similar economic characteristics to the UK. Future research could explore whether our findings persist across various geographical regions. In addition, this study examines the effect of overall IFRS adoption on the relationship between analyst coverage and REM. Future work may investigate the effect of a specific standard, or set of standards. Finally, this research focuses on the effect of the entencity of analysts following, future research may examine whether the characteristics of analysts, such as analyst experience, reputation, and forecast accuracy, affect earnings management.

## Notes

1 The monitoring role of the analyst may be exercised by organizing company visits, requesting clarification from management, and asking for access to top management.
^2^ Following Athanasakou, Strong, and Walker (2009), industries with fewer than six observations in each year were removed from the sample because of the lack of quorum in calculating the coefficient.
^3^ Running the regression for each industry in each year partially controls for industry-level changes in economic conditions, and allows the coefficients to vary across time (Doukakis, 2014).

## CRediT authorship contribution statement

**Mohammad I. Almaharmeh:** Writing – review & editing, Writing – original draft, Supervision, Software, Resources, Project administration, Methodology, Formal analysis, Data curation, Conceptualization. **Jia Liu:** Supervision, Conceptualization. **Majd Iskandrani:** Writing – review & editing, Visualization.

## Declaration of competing interest

The authors declare that they have no known competing financial interests or personal relationships that could have appeared to influence the work reported in this paper.

## Footnotes

1 IFRS Foundation. (2022). IFRS Foundation and GRI to align capital market and multi-stakeholder standards to create an interconnected approach for sustainability disclosures. Retrieved March 25, 2022, from IFRS Foundation website: https://www.ifrs.org/news-and-events/news/2022/03/ifrs-foundation-signs-agreement-with-gri/.
